# Nanotechnology strategy for inhibition of PARP1 and IL-17A-associated with neurotoxicity in rats exposed to hospital wastewater

**DOI:** 10.1007/s00210-024-03512-x

**Published:** 2024-10-18

**Authors:** Hend A. Sabry, Elham H. A. Ali, Amany A. Osman, Mai M. Zahra

**Affiliations:** 1 Zoology Department, Faculty of Women for Arts, Science, and Education, Ain Shams University, Cairo, Egypt; 2 Zoology Department, Faculty of Science, Ain Shams University, Cairo, Egypt

**Keywords:** Nanotechnology, HWW, PARP1, IL-17A, Behaviors, Neurotransmitters

## Abstract

Hospital wastewater (HWW) poses a serious hazard to human health security concerning its high susceptibility to neurodegeneration. Water sources and ecosystems are exposed to a complicated pollution load from a variety of refractory organics and pharmaceutical active composites. This study evaluates the treated newly developed nanocomposite (NiFe_2_O_4_) HWW on the neural injury induced by HWW action in rats. Three groups of male Wistar rats were distributed, with eight rats in each: group I: tap water served as a control; group II: HWW; and group III: nano-HWW. Each group was intragastrical administrated with each type of water (2.5 ml/100 g b.wt/6 h) for 28 consecutive days. The open field test and Morris Water Maze assessed behavioral activity and spatial learning 2 days before the last day. The research demonstrated that HWW treated with nanocomposite (NiFe_2_O_4_) may exert decreased risks of the neural impairment effect of HWW. This improvement was achieved by reducing the neurotoxicity by lowering nitric oxide contents, lipid peroxidation, acetylcholinesterase, interleukin-17A (IL-17A), and poly(ADP-ribose) polymerase1(PARP1) while restoring the antioxidant biomarkers and neurotransmitter levels (β-endorphin, norepinephrine, dopamine, and serotonin) of the treated groups in the cortex and brainstem and enhancement of the histopathology of the cortex as well. In conclusion, this study introduced a newly developed nanotechnology application for treating HWW to protect from neural injury. The findings of this research have significant value for policymakers, Ministry of Health management, and environmental organizations in their selection of suitable techniques and procedures to optimize hospital wastewater treatment efficiency.

## Introduction

Pharmaceutically active substances, including antibiotics and analgesics, make up a large portion of the new class of pollutants. Both aquatic and terrestrial ecosystems are vulnerable to the introduction of these pollutants, which endanger both humans and aquatic life. Patients eliminate these chemicals in a variety of forms, including the original molecules as well as metabolites and conjugates, which end up in sewage systems via urine and feces (Kanama et al. 2018). New pollutants found in low quantities (between micrograms per liter and nanograms per liter) have the potential to evade wastewater treatment facilities and end up polluting the environment (Kanama et al. 2018**)**. Half or even 70% of the new pollutants in HWW cannot be removed by conventional treatment procedures (Parida et al. 2022). High levels of organic debris, diseases, and new pollutants in aquatic environments are a result of wastewater discharged directly into the environment (Gupta and Gupta 2021). Lots of research has shown that hospitals especially in lower-middle-income nations dump their disinfectant, hand sanitizers formulated with alcohol, and pharmaceutical waste which includes antibiotics and resistant genes into the surrounding waterways without proper treatment (Parida et al. 2022**)**. Contamination of surface water, groundwater, soil, and sediments with these medications poses a risk to human and environmental health, and there may be neurological impacts on human health from long-term exposure to certain drugs, even at low levels (Khan et al. 2021).

In tandem with their extensive use, antibiotics have been continuously released into the environment via excretion. It is impossible for wastewater treatment facilities to eliminate all traces of antibiotics, and neither can people or animals metabolize them entirely. Consequently, the primary chemicals and their byproducts (metabolites) end up in the natural world. Urine and feces carry up to 70% of antibiotics that have not been digested (Yang et al. 2021). Conversely, antibiotics have a long history of usage in the treatment, control, and prevention of infectious illnesses in plants, animals, and people. Antibiotics are chemical agents that kill or restrict the development of microorganisms (Caracciolo et al. 2015). Thus, oxidative stress may be induced by medications such as analgesics and anti-inflammatory medicines (Sha et al. 2015). Additionally, reactive oxygen species may activate transcription factors that impact the production of pro-inflammatory cytokines, pro-oxidants, and antioxidants; this complicated feedback loop connects inflammation to the development of oxidative stress (Morgan and Liu 2011).

State of nutrition, localized flow of blood and tissue utilization, blood–brain barrier (BBB) condition, medicine absorption degree, mechanism of drug transport to the intended tissue, activation and clearance of the drug, protective responses, and medication metabolism all contribute to an individual’s neurotoxicity risk. Potentially important additional variables in neurotoxicity include genetics, renal insufficiency-related changes in drug pharmacokinetics, and penetration of the central nervous system (Grill and Maganti 2011). Effluent treatment is thus essential for hospitals to reduce their negative effects on the environment.

Nanotechnology offers several potential advantages for the treatment of HWW, one of which is the potential enhancement of the efficacy of existing treatment methods. One example is the potential use of nanoparticles in biological treatment procedures to facilitate the removal of contaminants and improve the overall efficacy of the treatment. In addition, treatment systems that use nanotechnology often have lower operating costs and use less energy as compared to traditional methods. One nanotechnology-based HWW treatment method is nanofiltration, which uses nanopores to filter out contaminants in wastewater using a membrane-based system (Hamad and El-Sesy 2023). Nanofiltration has the potential to extract organic waste and dissolved salts, among other small molecules. The use of nanoparticles with a high surface area in the adsorption process allows for the removal of contaminants from wastewater. This method has the potential to remove heavy metals, organic pollutants, and pathogens. Although using nanotechnology to treat HWW is promising, the release of nanoparticles into the environment poses serious risks. Researchers are still trying to figure out the pros and cons of using nanotechnology to remediate wastewater (Khan et al. 2019).

Due to the present complexities in HWW, its knowing treatments are not economical or effective. This is a pressing matter which needs a prompt response. The present article examines the effectiveness of nano-treatment of hospital wastewater in eliminating these medications on the neurotoxicity of rats. Through the application of magnetic NiFe_2_O_4_ nanocomposite, our research intends to remove and eliminate these pharmaceutical contaminants from hospital effluent (Mostafa et al. 2024). This treatment method eliminates a major threat to human health security due to its extreme vulnerability to neurodegeneration. So, the present study aims to evaluate this treated NiFe_2_O_4_ nanocomposite HWW effect on the brain of rats exposed to HWW by determining the biochemical, neurobehavioral, and histopathological protection of this nanocomposite.

## Material and methods

### Experimental animals

The National Research Center’s animal house was scouted for the acquisition of male albino Wistar rats weighing 120 ± 10 g. The experiment continued with the animals housed in standard polypropylene cages, with eight rats per cage. The animals were kept at a regulated ambient temperature (21 ± 2 ℃) and moisture of 55% with a 12-h dark–light sequence. Their acclimation to the lab environment began 7 days before the study. The rats were free access to food and fed on commercial neutral pallets. Ain Shams University Ethical Agreement Organization, under the sci1332403004 approval code, ensured that all animal research adhered to their standard rules for the upkeep and application of experimental animals.

### Sample collection

Wastewater from Ain Shams Specialty Hospital in north Egypt was collected before it was disposed of from the drainage pipes. During February and March of 2023, it was gathered. The samples were collected while the hospital was operating at full capacity (9:00 a.m. to 5:00 p.m.). Moreover, the consumption of water in all these instruments and analyzers, in addition to the test numbers, was used to estimate the water consumption that resulted from the primary hospital operations, including laboratory dialysis, sterilization, and analysis. Two large disposal points on the hospital grounds were used to collect the wastewater from all areas of the facility. This effluent was then combined and stored in a sterile 2-l glass container at 4 ℃ throughout this study.

### Preparation of NiFe_2_O_4_ nanocomposite

Aqueous solutions of ferric and nickel chloride were combined at a 1:2 molar percentage and then agitated continuously for 30 min. The pH was adjusted to 11 by gradually adding a solution of NaOH (2.0 M) to the mixture at a time of 1 mL/min. An hour of continuous stirring followed by heating to 100 ℃ resulted in a brown precipitate. The mixture was let to stand overnight to guarantee stability. Filtrate pH was brought down to 7.0 by rinsing the black precipitate with distilled water many times. The leftover material was gathered and then dried for 6 h at 110 ℃. After being crushed into a fine powder, the material was calcined at 600 ℃ for 5 h before being kept in a desiccator (Fathy et al. 2022). A fine powder was produced by crushing the material, which was heated up at 600 ℃ for 5 h and then stored in a desiccator.

### Characterization of nanocomposite

**Fig. 1:**
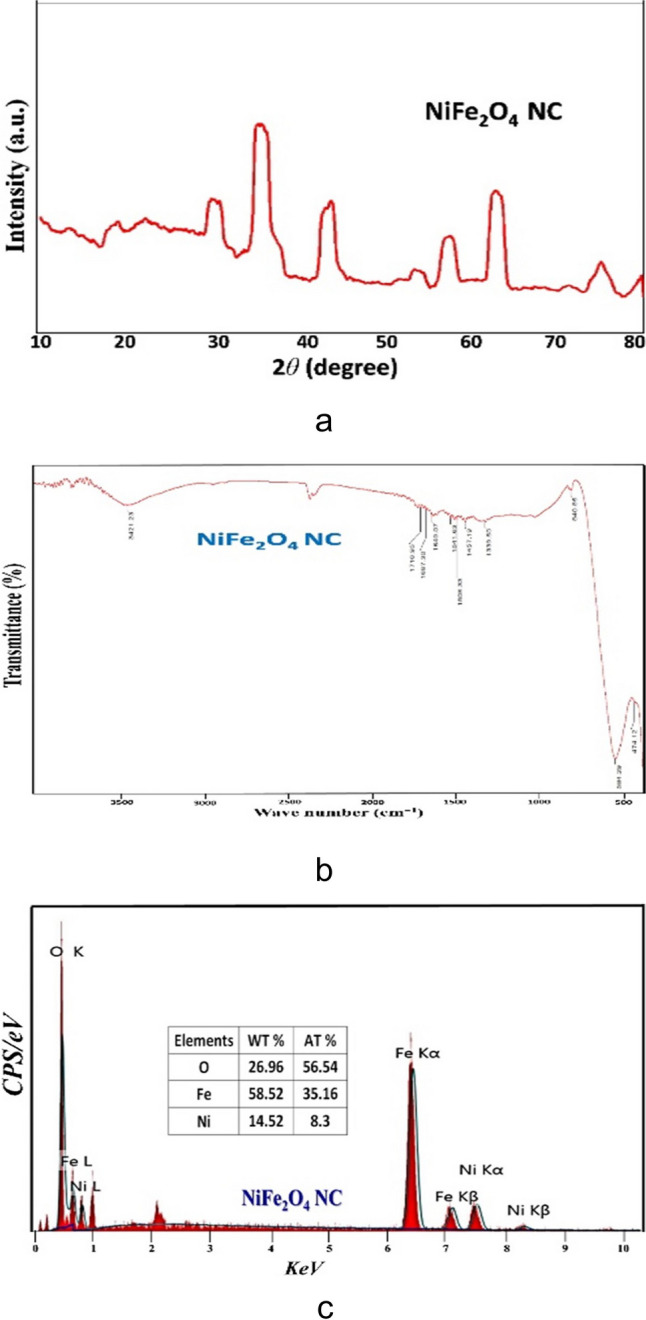
**a** X-ray diffraction (XRD) patterns, **b** Fourier-transform infrared (FTIR) spectra, and **c** energy dispersive X-ray (EDX) spectra of NiFe_2_O_4_ nanocomposites (published with permission from Mostafa et al. 2024)

**Fig. 2:**
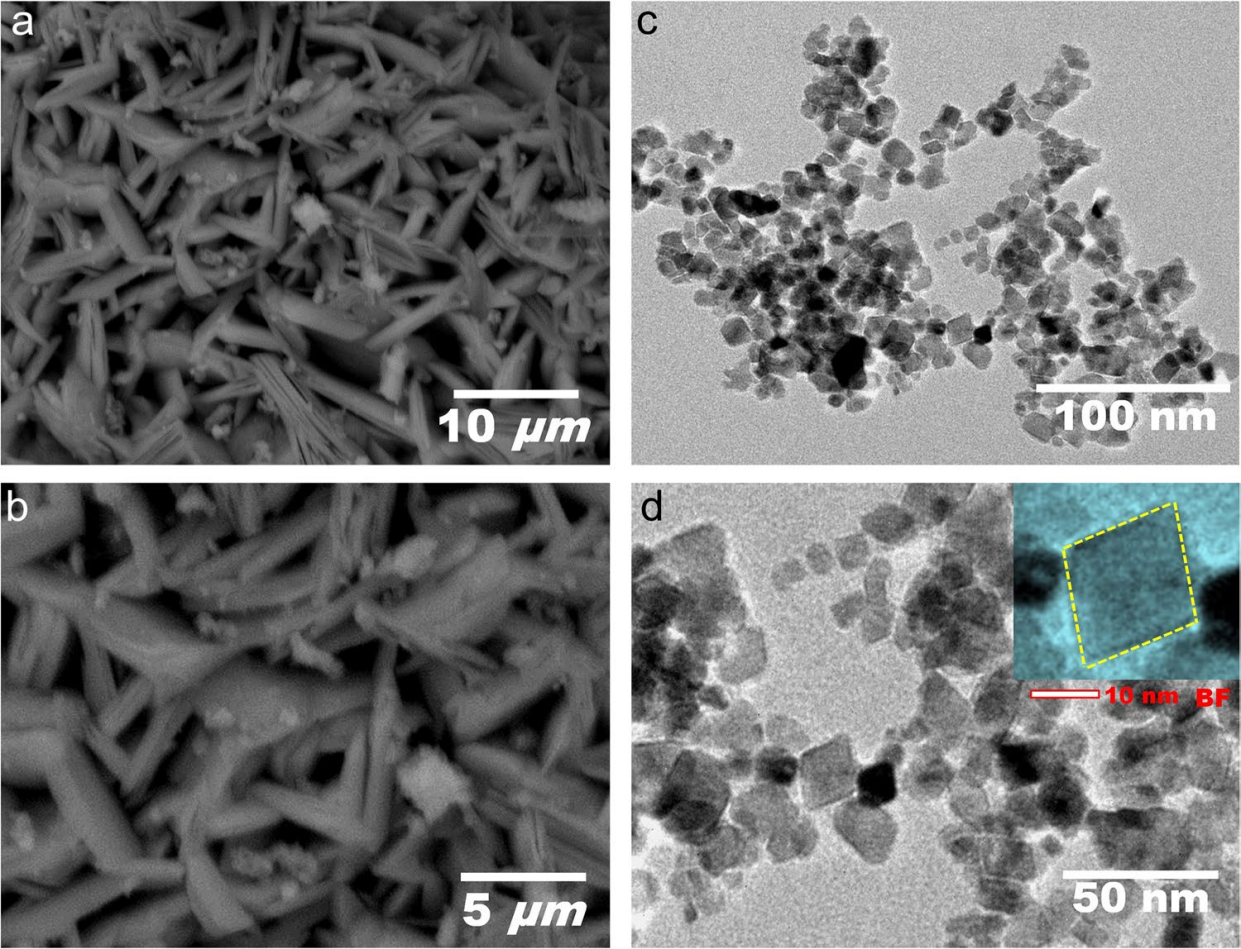
**a**, **b** Scanning electron microscope (SEM) and **c**, **d** transmission electron microscope** (**TEM) images of NiFe_2_O_4_ nanocomposite at various magnifications

As described in our previous study (Mostafa et al. 2024), measurements of XRD and X-ray diffraction were performed in the 2θ range (10–80 ℃) using Bruker AXS-D8, Germany, and CuKα radiation (*λ* = 1.5406 Å), a Pye-Unicam Sp-3–300 FTIR, Fourier-transform infrared, spectrometer, fitted with an 8101 PC and KBr method, was used to evaluate the infrared spectra; moreover, energy dispersive X-ray (EDX) analysis was also performed to confirm NiFe_2_O_4_ chemical (Fig. 1). In Fig. 2, the morphological descriptions were considered to NiFe_2_O_4_ using scanning and transmission electron microscopes (SEM, TEM). The JEOLJEM-1400EX TEM (Osaka, Japan) was used to capture the photographs. To disperse the powders within the samples for TEM studies, they were dissolved in ethanol. Subsequently, a droplet of the sample was carefully deposited onto a lacey carbon copper grid, which was utilized as a support for TEM analysis. After 2 h of drying at room temperature, the grid was subjected to reduced pressure. Twenty kilovolts were the TEM’s acceleration voltage.

### The treatment of HWW by nanocomposite

Subsequently, 1.0 L of HWW was combined with 0.1 g of nanocomposite (NiFe_2_O_4_), then the mix was agitated for an hour at ambient temperature using a magnetic stirrer. Then, for the experimental investigation, the treatment sample of HWW was filtered and preserved at − 20 ℃ until used (Mostafa et al. 2024).

### Composition of HWW

The composition of HWW was determined in our previous work by Mostafa and coworkers (2024), whose screen analgesics like diclofenac (DCF) and antibiotics (sulfamethoxazole, ofloxacin, amoxicillin, and oxytetracycline) and their concentrations in HWW using to the LCMS/MS and HPLC/UV, respectively.

### Experimental design

Correspondingly, the water consumption per day is 9 to 12 ml/100 g rat body weight (Claassen 1994). After a week of acclimation, each group was intragastrical administrated each type of water (2.5 ml/100 g b.wt/6 h for 28 consecutive days (Brahmi et al. 2024). Twenty-four rats were split into three groups for this experiment: the group served as the control group got regular tap water to drink, the second group got hospital wastewater (HWW), and the third group got HWW treated by nanocomposite, NiFe_2_O_4_ (nano-HWW).

### Assessment of behavior

#### Open-field test

This technique evaluates rats’ general locomotor activity and anxiety. It was 100 × 100 cm with 45 cm of walls, made of white plywood. Using a marker, blackened outlines were painted on the ground. The floor was split into twenty-five 20 × 20 cm^2^ areas by the lines. In the middle, a 20 cm by 20-cm middle area was squared. Two days before the end of the experiment, each rat was put into one of the field’s four corners and assumed 5 min investigating the device. Rats were returned to their home enclosures following the assessment. The device was cleaned with ethyl alcohol (70%) and dried among trials. According to Sabry and Zahra (2024), on two different days, rats were put in the maze for 5 min each to assess their behaviors. The statistics assumed the advantage of the next-day trials’ observation, which was documented.

#### Morris Water Maze (MWM)

Researchers looked at rats’ mental and cognitive abilities with the MWM test. It is a wide container ~ 1.2 m in diameter and 0.45 m in height filled with water at a temperature of 27 ℃ up to a depth of 0.35 M. The tank was divided into four subdivisions to accommodate the rats’ spatial orientation needs. Each subdivision had fixed and various distal room signs, which the rats could see from the pool. A concealed black platform measuring 0.14 m in diameter and 0.34 m in height was positioned within the target quadrant of the pool, 0.1 m beneath the water’s surface. It is perpendicular to the pool’s border with constant placement throughout the experiment. The animal underwent four trials daily for two consecutive days. The rat could swim for up to 60 s before finding the platform and exiting the water. Being on the platform for not less than 10 s is generally characterized as discovering it. If the rats did not discover the platform within the 60 s, the rat was physically placed on it and allowed there for 10 s. In a comparable vein, if rats independently discovered the platform, they may remain on it for 10 s, and the number of crossings over the site of the platform was recorded (during hidden platform training, the platform was set up) (Ali and Arafa 2011).

### Tissue collection and preparation

The experimental animals were slaughtered via quick decapitating after the next day performing an open field test. Following dissection, each of the six brains was split in half lengthwise. After removing the cortex and brainstem from the first half, they were homogenized in a phosphate buffer (0.05 M, pH 7). The homogenates were centrifuged at 5000 rpm. for 5 min at 2–8 ℃. The high-performance liquid chromatography (HPLC) analyzes the supernatant, along with a colorimetric test. Enzyme-linked immunosorbent assay (ELISA) was conducted consuming the supernatant of homogenizing the second half of the brain in the same buffer. For histological analysis, the two remaining rat brains were separated.

### HPLC technique

The HPLC/UV method was used to quantify the levels of neurotransmitters (β-endorphin, dopamine (DA), norepinephrine (NE), and serotonin(5HT) in brain cortex and brainstem regions according to Pagel et al. (2000). Under these requirements pH 3.0 potassium phosphate mobile phase, 1.5 ml/min current rate, and 270 nm UV, the supernatant was directly injected onto an AQUA column 150 × 4.6 mm 5 µ C18. There was a 10-min separation of DA, NE, and 5HT. By comparing the sample with the standard, the chromatogram revealed the relative positions and concentrations of each monoamine.

### Colorimetric assay

The thiobarbituric acid reactive substance (TBARS) test, which was reported by Draper and Hadley (1990), was used to determine the concentration of malondialdehyde (MDA) at a wavelength of 532 nm, which was employed as a marker of lipid peroxidation index. Using a BIODIAGNOSTIC kit, nitrite was used to characterize nitric oxide (NO) at 540 nm wavelength. Prins and Loose’s (1969) method of measuring reduced glutathione (GSH) activities involved protein precipitating by the solution of tungstate-sulfuric acid, and then seeing the yellow development color at a wavelength of 412 nm afterward, the reaction through 5,5 dithiobis-25-nitrobenzoic acid. At 340 nm wavelength, the activity of glutathione S-transferase (GST) was determined with CDNB,1-chloro-2,4-dinitrobenzene like a base (Habig et al. 1974). Böck et al. (1980) method utilized catalase-induced hydrogen peroxide breakdown, which was used to determine the catalase (CAT) time at a wavelength of 240 nm. Superoxide dismutase (SOD) activity was estimated at 540 nm wavelength with Nishikimi et al. (1972) procedure outline. The enzyme’s capacity was used to obstruct phenazine methosulphate’s ability to reduce nitroblue tetrazolium dye. The colorimetric estimation of the activity of AChE, acetylcholinesterase, was performed (Gorun et al. 1978). Specifically, acetylcholinesterase hydrolyzes acetyl-thiocholine forming thiocholine. It was reacting with DTNB, 5,5-dithiobis-2-nitrobenzoic acid, to give yellow 5-thio-2-nitrobenzoate, which can be measured photometrically at 412 nm wavelength.

### Enzyme-linked immunosorbent assay

Procedures for detecting interleukin-17A (IL-17A) and PARP 1, poly-ADP-ribose polymerase-1, using an ELISA in combination with guidelines provided by the manufacturer. The ELISA kits for IL-17A and PARP 1 were acquired from Elabscience and Life Span BioSciences, respectively, using the following codes: E-EL-R0566 and LS-F27548, respectively. The plate reader of ELISA measured the absorbance color of IL-17 and PARP 1 within an optical density range of 450 nm (Notice Technologies, Florida, USA, sends Stat Fax 2200.).

### Histological examination

Specifically, Bancroft and Stevens (2016) lay out the steps to get histology samples. In short, the remaining brain tissue was sectioned into slices that were thick (3–4 mm) and then restored in neutral-buffered formalin (10%). It was then dried utilizing ethyl alcohol at variable ratios, washed in xylene, then inserted finally in the paraffin. To analyze all structures of tissues, the blocks of paraffin were sliced into slices via the microtome at 4–6-μm thickness and then dyed with hematoxylin and eosin. The Leica microscope (CH9435 Hee56rbrugg) from Leica microsystems in Switzerland analyzes sections tainted with hematoxylin and eosin.

### Statistical analysis

For this statistical study, we consulted SPSS, Chicago, IL, Statistical Package for the Social Sciences, Version 23.1 for Windows. The data presented as means ± standard errors of six and eight rats intended for biochemical and behavioral examinations, respectively. The alterations among the various groups were assessed once *F* test significantly has been attained with realizing comparisons of the LSD (least significant difference) at *P* < 0.05.

## Results

### Results of biochemistry

The results from Table 1 demonstrated a notable rise in oxidative stress due to the HWW treatment. This was confirmed by the fact that the contents of nitric oxide (NO), malondialdehyde (MDA), and acetylcholinesterase (AChE) of the brain cortex and brainstem were increased. At the same time, the activities of glutathione S-transferase (GST), reduced glutathione (GSH), superoxide dismutase (SOD), and catalase (CAT) were decreased with comparison to the control group, with significant levels at *P* < 0.05. There were substantial decreases in cerebral cortex and brainstem MDA, NO levels, and AChE activity in the treated group with nano-HWW when compared to the group of HWW. On the one hand, when contrasted with the HWW group, nano-HWW therapy resulted in a significant upregulation of GST, GSH, and CAT activities in the brainstem at *P* < 0.05. On the other hand, cortex and brainstem MDA contents and CAT activities were significantly decreased in nano-HWW when compared to the group of control, whereas GSH levels and AChE activities increased significantly in the nano-HWW group in comparison with.

**Table 1:** The effect of hospital wastewater (HWW) and nano-HWW on nitric oxide (NO, µmol/g tissue), malondialdehyde (MDA, µmol/g tissue) contents, glutathione S-transferase (GST, mmol/min/g tissue), reduced glutathione (GSH, mg/g tissue), superoxide dismutase (SOD, µg/g tissue), catalase (CAT, mmol/min/g tissue), and acetylcholinesterase activities (AChE, µg/g tissue)

	Cortex			Brainstem		
	Control	HWW	Nano-HWW	Control	HWW	Nano-HWW
NO	0.34 ± 0.04	0.59^*^ ± 0.04	0.35^#^ ± 0.02	0.36 ± 0.03	0.69^*^ ± 0.09	0.39^#^ ± 0.02
MDA	1.74 ± 0.13	6.03^*^ ± 0.26	2.43^#^ ± 0.1	2.07 ± 0.28	4.96^*^ ± 0.16	3.17^*#^ ± 0.11
GST	6.59 ± 0.49	4.56^*^ ± 0.78	6.44^#^ ± 0.43	7.32 ± 1.01	4.84^*^ ± 0.28	6.44 ± 0.98
GSH	4.07 ± 0.26	1.19^*^ ± 0.07	3.35^*#^ ± 0.21	4.17 ± 0.23	2.64^*^ ± 0.21	3.04^*^ ± 0.28
SOD	1.89 ± 0.07	1.51^*^ ± 0.08	1.60 ± 0.07	1.77 ± 0.06	1.54^*^ ± 0.07	1.63 ± 0.07
CAT	13.66 ± 0.31	8.11^*^ ± 0.63	10.14^*^ ± 1.09	13.52 ± 0.48	7.47^*^ ± 0.74	10.21^*#^ ± 0.63
AChE	2.11 ± 0.04	3.31^*^ ± 0.18	2.66^*#^ ± 0.04	1.51 ± 0.02	3.83^*^ ± 0.11	2.42^*#^ ± 0.17

The values given are the mean ± SEM of six rats in each group. With a *P*-value < 0.05, * means a significant change from the control group and # means a significant change from the HWW group

**Fig. 3:**
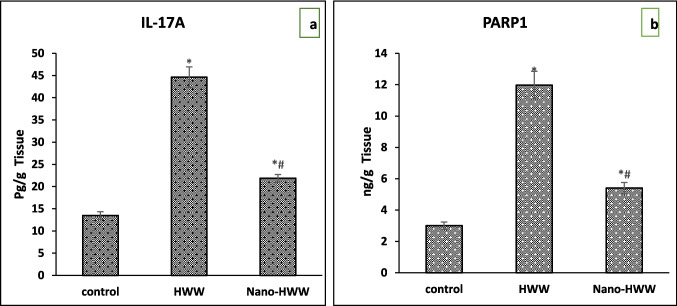
**a**, **b** Effect of hospital wastewater (HWW) and nano-HWW on **a** interleukin-17A (IL-17A) and **b** PARP 1, poly-ADP-ribose polymerase-1, later 28 days of treatment. Values given are the mean ± SEM of six rats in each group. With a *P*-value < 0.05, * means a significant change from the control group and # means a significant change from the HWW group

In whole brain tissue, Fig. 3a, b indicates that levels of IL-17A and PARP1 increased significantly in treatment groups compared to the control group. The nano-HWW group demonstrated a substantial reduction in IL-17A and PARP1 levels significantly at *P* < 0.05, in contrast to control and HWW groups, which did not exhibit any noticeable improvement.

**Fig. 4:**
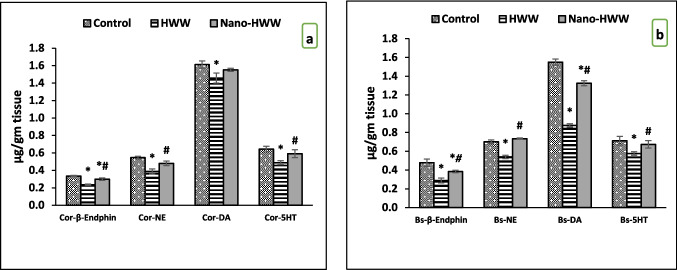
**a**,** b** Effect of hospital wastewater (HWW) and nano-HWW on neurotransmitter contents (β-endorphin, dopamine (DA), norepinephrine (NE), and serotonin (5-HT) in **a** brain cortex and **b** brainstem later 28 days of treatment. Values given are the mean ± SEM of six rats in each group. With a *P*-value < 0.05, * means a significant change from the control group and # means a significant change from the HWW group

These results shown in Fig. 4a, b indicated that there was a substantial drop in cortex and brainstem levels of neurotransmitters (β-endorphin, NE, DA, and 5 HT) in the treated groups with HWW in comparison with the control group. In contrast to the group treated with HWW, the treated group with nano-HWW showed a notable rise in the β-endorphin, 5 HT, and NE levels. Additionally, as a comparison of the groups treated with nano-HWW to the HWW-treated group, all neurotransmitter contents increased significantly in the former (*P* < 0.05), whereas the cortex in addition to brainstem β-endorphin levels and brainstem DA was significantly decreased in nano-HWW-treated groups when compared to the control group.

### Behavioral results

**Fig. 5:**
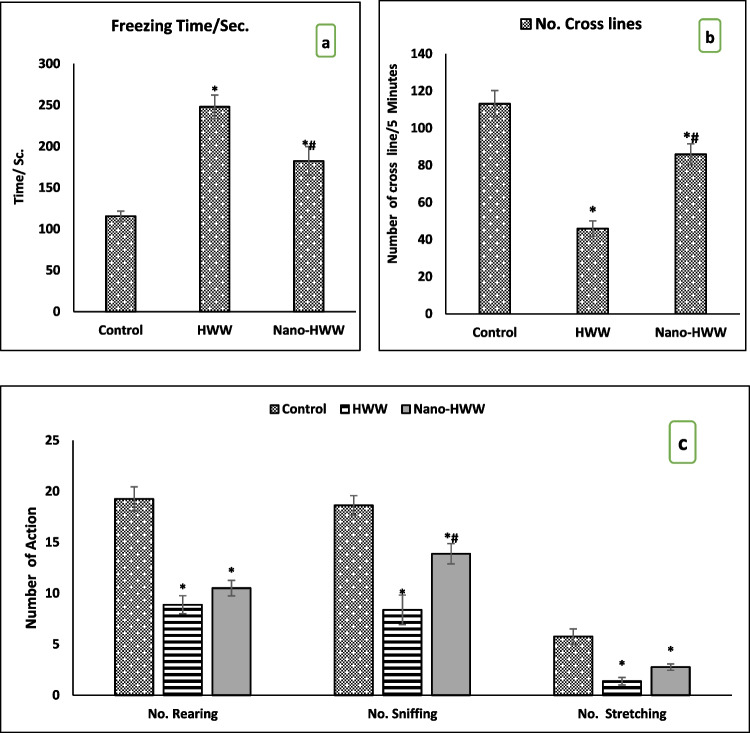
**a**–**c** Effect of hospital wastewater (HWW) and nano-HWW on open field test **a** freezing time (Sc)/5 min, **b** crossing line movement number/5 min, and **c** rearing, sniffing, and stretching/5 min later 28 days of treatment. Values given are the mean ± SEM of eight rats in each group. With a *P*-value < 0.05, * means a significant change from the control group and # means a significant change from the HWW group

In comparison with the control group, all the treatment groups showed a significant increase in freezing time and a decrease in movement crossing lines number after 28 days of treatment, according to the open field test results presented in Fig. 5a, b with *P* < 0.05. As compared to the group treated with nano-HWW-treated to the group treated with HWW, there was a significant difference in the freezing time and crossing lines number. The frequency of rearing, sniffing, and stretching behaviors was decreased significantly in all treatment rats as compared with control rats, as shown in Fig. 5c. The stretching and rearing numbers performed by the group treated with nano-HWW did not increase significantly when compared to the group treated with HWW. Conversely, there was a substantial increase in sniffing in the nano-HWW-treated group compared to the HWW-treated group at *P* < 0.05.

**Fig. 6:**
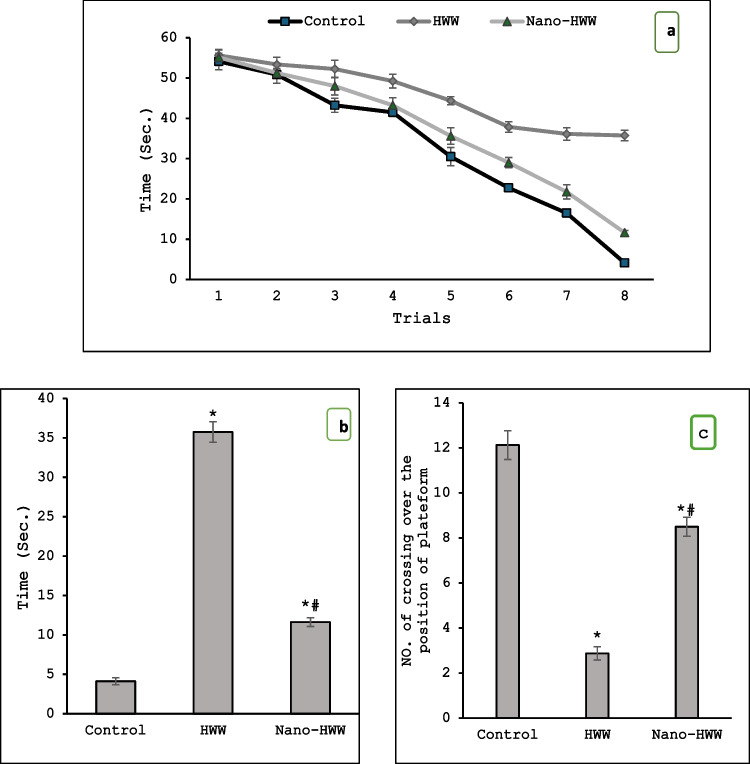
**a**–**c** Effect of hospital wastewater (HWW) and nano-HWW on Morris Water Maze (MWM) test after 28 days **a** approaching the eight trials of the MWM test, **b** expressing the expectancy time (eighth trial), and **c** the number of crossings over the platform position trial (retention trial). Values given are the mean ± SEM of eight rats in each group. With a *P*-value < 0.05, * means a significant change from the control group and # means a significant change from the HWW group

The rats treated with HWW or nano-HWW for 28 days were exposed to a significant increase in the expectancy time to access the obscure platform in Fig. 6a, b with significant decreases in learning and memory functions in the number of crossings over the platform position (memorizing trial) as competed to the control group (Fig. 6c) at *P* < 0.05. Concerning MWM outcomes, the nano-HWW group revealed a significant decrease in expectancy time in comparison with the HWW group at *P* < 0.05 (Fig. 6a, b). Furthermore, the nano-HWW treatment adjusted the down memory retention significantly in comparison to the HWW group (Fig. 6c).

### Histological and histopathological results

**Fig. 7:**
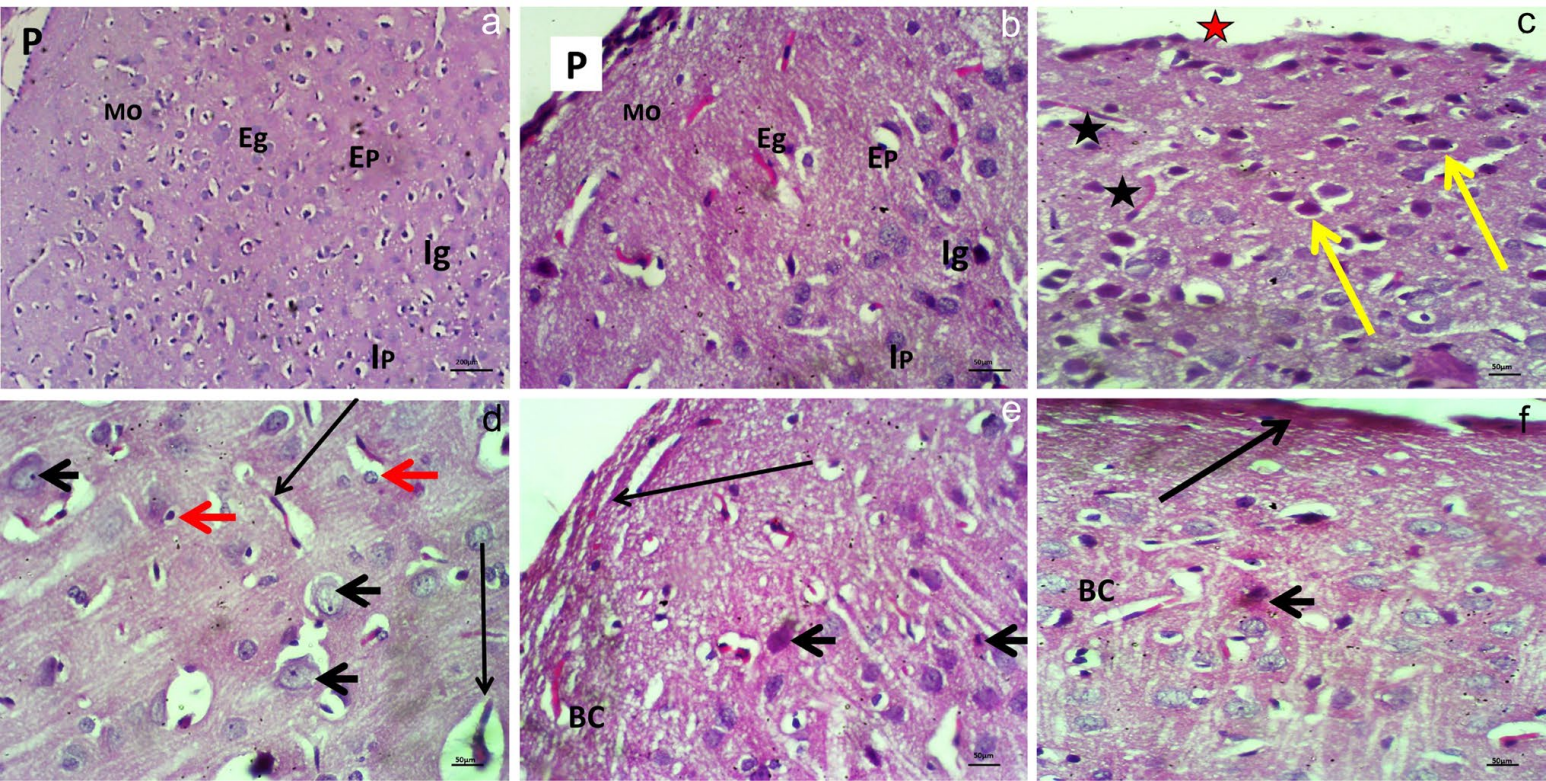
A photomicrographs of cerebral cortex tissue in **a**, **b** the control group revealed normal coordination structure of nerve tissue cells, showed molecular layer (mo) was protected with regularly attached pia matter (P) rich by blood capillary (stars) with peripheral dilated one (red star) (BC), exterior granulated layer (Eg), exterior pyramidical layer (Ep), interior granulated layer (Ig), interior pyramidical layer (Ip) (HE X200, 400) **c** the cerebral cortex tissue in HWW group illustrate hyperchromatic appearance (arrows), other erosion in pia matter layer (red star)—invasion of dilated expended blood vessels (BV) (black star) (HE X400), and **d** other magnified field showed vacuolar hydropic change accompanied by pyknotic nuclei (arrow heads) invasion dilated expended BV (long arrow) shrinkage in glial and astrocytes cells (red arrows) that affected its function (HE X400), **e**, **f** in the nano-HWW group showed some extent of the cortex shows a noticeable improvement, with slight congestion of some BC, some nuclei suffering from condensation of nuclear material (arrow head) and a slightly destabilization of the structure of the pia mater layer (long arrow) (HE X400)

In Fig. 7a, b the control group’s cerebral cortex histological structure demonstrated normal coordination structure of nerve tissue cells, with the molecular layer covered in regularly attached pia matter rich in blood capillaries, exterior granulated layer, outer pyramidical layer, interior granulated layer, and peripheral dilated one (HE X200, 400). In Fig. 7c, the HWW group’s cerebral cortex tissue has a hyperchromatic appearance, which may be caused by a shift in nuclear mass. An increased nuclear/cytoplasmic ratio may also result in neoplastic changes, additional erosion of the pia mater layer, and invasions by dilated, enlarged blood vessels (HE X400). Other enlarged fields (Fig. 7d) revealed vacuolar hydropic alteration in glial and astrocyte cells along with pyknotic nuclei invasion, dilated exhausted blood vessels shrinking, and other changes that impacted its function (HE X400). Figure 7e, f, a considerable improvement was seen in the cortex in the nano-HWW group, with some capillaries showing minor congestion, certain nuclei experiencing nuclear material condensation, and a small instability of the pia mater layer structure (HE × 400).

## Discussion

Hospital wastewater (HWW) has a complicated chemical makeup and a high potential to spread illness, making it hazardous to both the environment and human health. Ofloxacin (OFX) and diclofenac (DCF) were more prevalent among the various chemical compounds found in the HWW sample (Vieira et al. 2021; Khan et al. 2022). According to Ngigi et al. (2019), Aleksić et al. (2022), and McCarthy et al. (2021), major antibiotics used in human medicine that have low biodegradability and significant environmental toxicity are sulfamethoxazole (SMZ), amoxicillin (AMX), and oxytetracycline. Heavy metals found in HWW pose a risk to human and environmental health due to their ability to bioaccumulate and bio-magnify. When it comes to people and the planet, various heavy metals have varied effects (Sakina et al. 2023). They have been found in various hospital effluents. Moreover, our previous study (Mostafa et al. 2024) found all the former pharmaceutical compounds in the HWW used in the present work.

According to McCarthy et al. (2021), both stabilized and unstabilized effluents, depending on their metabolism, enter the urban wastewater network untreated. All living things, including people, are vulnerable to a wide range of biological and environmental hazards. Harmful and poisonous byproducts, free radicals, and oxidant species contribute to cellular and organ malfunction. Damage to DNA, lipids, and proteins might occur from an excess of these species. However, physiological reactions include signaling and defense systems that include low to moderate extents of RNA, reactive nitrogen species, ROS, and reactive oxygen species. One of the most prevalent ways in which environmental pollutants cause harm is oxidative stress (El-Demerdash et al. 2018). Degenerative brain disorders like Alzheimer’s and Parkinson’s are associated with pathological alterations brought on by an imbalance in the antioxidant defense system and an unchecked accumulation of species ROS in the tissue of the brain (Singh et al. 2019**)**.

Neri-Cruz et al. (2015) observed the increase in oxidative stress biomarkers and disturbance in antioxidant activities in carp *Cyprinus carpio* associated with HWW induction for 1, 2, 3, and 4 days in Mexico. Islas-Flores et al. (2013) explained the observed changes in the current study’s HWW treatment group related to the group of control, including an enhancement in peroxidation of lipid and nitric oxide levels, a decline in antioxidant activity, in addition to inflammation response. As demonstrated in our earlier research, one of the pollutants found in HWW is the analgesic biological transformation in humans (Mostafa et al. 2024**)**, four hydroxydiclofenac is produced by CYP2C9 hydroxylation and is structurally identical to DCF. In the process of DCF hydroxylation metabolism by the P450 complex, many investigations have shown that various organ systems may create ROS, including the superoxide anion radical (O_2_*) (Hoeger et al. 2008). Thus, DCF and other NSAIDs influence the mitochondria and oxidative phosphorylation, which might lead to an increase in ROS generation.

Evidence suggests that many DCF metabolites bind to proteins and suppress their action, leading to decreased SOD and CAT activities in the brain of carp *Cyprinus carpio* (Saucedo-Vence et al. 2015). Like this, antibiotics work against microorganisms by causing oxidative damage in bacteria, which is frequently followed by the release of ROS. Since proteins frequently function as enzymes in organisms, they are more vulnerable to reactions from these reactive species than lipids, deoxyribonucleic acid, or other biomolecules. Notably, every single one of the plasma thiol groups connects to a protein, and they are oxidatively sensitive indices used in the defense mechanism against reactive oxygen species (Oyewo et al. 2021**)**.

Additionally, the BBB disturbance (decreases occludins, claudins, and zonula occludens) plus the reduction in AchE activity showed brain damage caused by exposure to the antibiotic sulfamethoxazole (SMZ). By increasing MDA and 8-OhdG, 8-hydroxy-2 deoxyguanosine, levels, and glutathione inhibition, SMZ caused oxidative stress, which in turn raised the intra-nuclear NF-κB level and its objective genes (interleukins and TNF-α), resulting in microenvironmental inflammation (Wang et al. 2022).

In the present study, SMZ, ofloxacin, and AMX were reported in the analysis of HWW by Mostafa et al. (2024). Reactive oxygen species overproduction caused DNA damage when ofloxacin was used, additionally, decreasing the CAT, SOD, and glutathione peroxidase (GPx) activities (Li et al. 2009). Moreover, AMX and its primary breakdown product AMX acid in HWW increase lipid peroxidation, hydroperoxide content, and protein carbonyl content in addition to considerable changes in antioxidant enzymes activity SOD, CAT, and GPx (Elizalde-Velázquez et al. 2017). In rats that were given oxytetracycline, Kalimuthu (2012) found that lipid peroxidation indicators (TBARS and lipid hydroperoxides) were significantly higher, whereas levels of both enzymic and non-enzymic antioxidants, including GSH and vitamins (C, E) were shown to be minor. Research has demonstrated that heavy metals can attach to sulfhydryl groups, causing harm to the structures that have these molecules. Mercury can reduce glutathione (GSH) levels and cause the production of ROS either directly or indirectly. Cadmium cannot directly generate ROS generation but can modify GSH levels and impact cellular thiol status, causing metallothionein expression. Nickel toxicity is linked to the reduction of GSH levels and its interaction with protein sulfhydryl groups, which can impair protein function as well as structure (Neri-Cruz et al. 2015).

In the current experiment, the increasing IL-17A level in the HWW group may be discussed by Youwakim et al. (2023) who postulate that IL-17A neuro-impairment is mostly mediated by increased superoxide anion generation. Peroxynitrite, a highly reactive oxidant, forms when superoxide anions react with nitric oxide. Microglia key immune cells in the central nervous system are considered the brain’s tissue macrophages. An essential reaction to neuroinflammation is microgliosis, which is characterized by an increase in the number of microglia. Overexpression of IL-17A in astrocytes was also shown to activate microglia, and neurodegenerative illnesses and cognitive impairment have been validated (Chen et al. 2020).

Also, increasing PARP-1 in our HWW data was described as inflammatory activities, which are commonly characterized by an unregulated increase in the amounts of ROS and nitrogen species, which cause DNA damage. Therefore, DNA repair necessitates an upregulation of PARP-1 activity. Consequently, PARP-1 hyperactivation can trigger parthanatos a planned caspase-independent death of cells (Maluchenko et al. 2021). Massive amounts of PARylated and poly-ADP-ribose proteins move from the nucleus to the cytoplasm, where they trigger the release of apoptosis-inducing factors from the mitochondria. This factor is then activated by endonucleases and returns to the nucleus through a nuclear location signal, leading to fragmentation of DNA and cell death (Mashimo et al. 2021). Additionally, to research by Mekhaeil et al. (2022), when PARP-1 is over-activated, it clues to a reduction in NAD + , nicotinamide adenine dinucleotide, and levels of ATP, which in turn causes cell death.

David et al. (2018) found alterations in glutamate, tryptophan, serotonin (5HT), and acetylcholine levels in altered brain areas of fish exposed to wastewater sewage. In the current work, drinking HWW depletes dopamine (DA) norepinephrine (NE), and 5 HT, as mentioned in several papers, that link neuronal death to oxidative stress, elevated the turnover rate of monoamines and low glutathione content (Liedhegner et al. 2011).

Therefore, more oxidative stress can harm the neurons that have survived. When exposed to diclofenac, the dopamine, and its derivative, DOPAC (3, 4-dihydroxyphenylacetic acid) levels in the brain of the *Rhamdia quelen* fish were shown to decrease (Guiloski et al. 2017). Additionally, the immobility time, swimming duration, and serotonin levels of brain tissues were all shown to be lower after AMX treatment (Atli et al. (2016). Furthermore, when microglia were present, IL-17A made the death of dopaminergic neurons much worse (Liu et al. 2019).

Some of the other ways that heavy metals can harm the nervous system include interacting with micronutrients like iron, zinc, and copper, which have been linked to attention deficit hyperactivity disorder (ADHD), and by causing lipid peroxidation and oxidative stress in the brain (Huang et al. 2023). Several pathways exist via which neurotoxic metals (Cd, Mn, As, Pb, and Hg) can interact via receptors of neurotransmitters. These include, but are not limited to, changes in protein and gene expression, and disturbance activities indirectly caused by ROS (Carmona et al. 2021). They imply that early exposure to Mn decreases DA levels, which in turn upregulates DA-D2 receptor expression dysregulates DA-associated signaling pathways, and depresses presynaptic dopaminergic function. There were several negative neurobehavioral effects associated with metal exposure in the HWW group, including a marked decrease in tyrosine hydroxylase expression and an increase in dopamine and serotonin reuptake transporter expression (Pyatha et al. 2023). Finally, it has been suggested that metal exposure can disrupt neurotransmitter biosynthesis.

Some dangerous medications, including streptozotocin and ampicillin, upset the delicate equilibrium of intestinal bacteria (Zarrinpar et al. 2018). Chemical substances or the nervous system can cross the blood–brain barrier to establish a connection between the microbes of the gut and the central nervous system. Specifically, the vagus nerve links neurons in the intestines to those in the brain (Angelucci et al. 2019). Substances (such as monoamines and amino acids) are produced by the gut microbiota. Antibiotics worsened spatial memory and heightened anxiety-like behavior (Wang et al. 2015).

Also, neuroinflammation is mostly caused by increased levels of proinflammatory cytokines, as shown by Angelucci et al. (2019), where the persistent Aβ peptide accumulation in the extracellular space appears to be the cause of microglia and astrocytes’ production of cytokines. The clinical symptoms of neurodegenerative disorders are caused by these deposits, which cause synaptic dysfunction, according to the amyloid cascade theory. According to Ceylani et al. (2018), antibiotics altered the microbiota of the intestines in ways that were linked to hormonal changes, including altered expression of BDNF, oxytocin, and vasopressin, a decline in adult hippocampal neurogenesis, and modifications to anxiety-like reactions, exploratory behavior, and cognitive abilities. Also, heavy metal exposure like arsenic has been linked to neural behavior symptoms, depression, attention difficulties, anxiety, and decreased intellectual development (Chu et al. 2023). The interpretation with the present study is possible, where the HWW contains antibiotics, heavy metals, and chemical compounds that harm gut microbiota which induced a significant increase in IL-17A and decreased neurotransmitters that reflected also on the behavioral action of the rats treated with HWW.

Pharmaceutical products sufficient to amend neurotransmitter levels in addition to harm economically appropriate behaviors in male goldfish *Carassius auratus* exposed to plants treated with wastewater sewages for 3 weeks (Simmons et al. 2017). This disturbance in neurotransmitters may explain the decrease in locomotion and activity with increasing anxiety which was demonstrated using an open field test in the present study, where DCF and antibiotics significantly intensified spatial memory impairment and depletion degrees of learning in the MWM test (Mosaferi et al. 2021). In addition, researchers found that the CdCl2 group spent much less time in the target quadrant and had a much longer time to escape in the same test. Also, the CdCl_2_ group exhibited longer periods of immobility and freezing when tested in open fields (Adebiyi et al. 2022). Conversely, mice lacking PARP-1 showed signs of anxiety, sadness, impaired communication, cognitive decline, and deficiencies in prepulse inhibition (Hong et al. 2019). Furthermore, Alves de Lima et al. (2020) proposed that anxiety is influenced by the physiological discharge of IL-17A at the meningeal–brain connection.

Also, in the current investigation, the tissue damage was assessed using histological testing, which revealed changes to the cerebral cortex’s histoarchitecture. Analogously, the brain appeared elaborated pyramidic cells, vacuolation, binucleated nuclei, and necrosis following exposure of fish *Oreochromis mossambicus* to the wastewater for 3 weeks (Navaraj and Yasmin 2012). According to Awad et al. (2022), oxidative stress-induced damage to cells can occur with an inequality between the formation of reactive oxygen species and the defense mechanism of antioxidants. When ROS attack cellular molecules and the plasma membrane, they destroy DNA and induce lipid peroxidation, which disrupts cells and injures tissue. Maluchenko et al. (2021) discovered that PARP-1 hyperactivation contributes to a series of processes set off by β-amyloid peptides, the build-up of which results in brain cell death. The cerebral cortex showed a substantial upsurge in PARP-1 expression and PAR polymer buildup. The least preferred type of cellular death is necrosis because it releases the contents of the cell to the tissue, exposing nearby cells to proteases and other proinflammatory agents and starting a positive feedback loop that leads to inflammatory tissue damage. Moreover, intracerebral hemorrhage is associated with neuroinflammation and microglial activity mediated by IL-17A (Yu et al. 2016).

Of the newly recognized pollutants (Ecs) in the HWW, only 50–70% can be eliminated by conventional treatment techniques. However, a lot of nations still lack appropriate in situ treatment protocols for HWW (Parida et al. 2022). This study’s treatment of HWW with nanoparticles improved their antioxidant activity while decreasing lipid peroxidation, nitric oxide, IL-17A, PARP1, and neurotransmitters. Additionally, the histopathology of the brain cortex improved, which may have been caused by the HWW’s loss of a significant proportion of chemical and pharmaceutical compounds. In the present study, NiFe_2_O_4_ nanocomposite, FTIR, XRD, SEM, and TEM characterization results revealed that the nanoparticles have a uniform, cubic structural morphology with distribution clusters with ordinary size ranging from 13.8 to 16.7 nm and thin size provinces between 2 and 6 nm in TEM, as described in Mostafa et al. (2024). Earlier work illustrated that NiFe_2_O_4_ exhibits a specific surface with a cluster of minor elements that consisted of insertions and protrusions which increase the adsorbent area (Shao et al. 2012; Alamier et al. 2023). Thus, the antibiotics SMZ, ofloxacin, oxytetracycline, and AMX derivatives were effectively eliminated by an efficacy of around 88% in under 40 min at equilibrium (Mostafa et al. 2024).

In the efficient treatment of a simulated pharmaceutical effluent for analgesics within 60 min, the NiFe_2_O_4_/activated carbon magnet complex material achieved a removal rate of 95%. Also, utilizing magnetized nanoparticles of nickel ferrite, Mostafa et al. (2024) found that DCF adsorbed at its maximal efficiency. Moreover, it effectively converted SMZ into a variety of compounds, some of which may 1 day produce basic ions and mineral acids (Nawaz et al. 2020; Makofane et al. 2022). The use of NiFe_2_O_4_ as photographic catalysts allows for the removal of impurities while simultaneously leaving behind high thermodynamic energies, which can speed up redox processes that break down medicines like ofloxacin and oxytetracycline (Zhao et al. 2021; Song et al. 2023).

Furthermore, Derakhshani et al. (2023) demonstrated that a magnetized nanocomposite of NiFe_2_O_4_ had a high maximum capacity for penicillin G, ampicillin, and AMX derivative adsorption. One of the most successful ways to remove heavy metal ions from wastewater is using nanotechnology filters. These membranes are affordable, versatile, and operate efficiently (Covaliu-Mierlă et al. 2023). Additionally, the nanoparticle of NiFe_2_O_4_ has the potential to replace the costly adsorbents used to eliminate divalent ions of heavy metal from contaminated water, and the structure of Langmuir isotherm and dynamical model of pseudo-second order with a monolayer capability for adsorption were shown to be good fits for the adsorption process (Ahmed et al. 2021; Mostafa et al. 2024).

## Conclusion

This study introduces a possible solution for one of the most prevalent water problems, of HWW using NiFe_2_O_4_ nanocomposite. Studies on the effect of HWW on the brain and behaviors are scarce and still need more research. For humankind’s multifaceted neuroprotection, social progress, and economic prosperity, clean and safe water is an essential need. Nanocomposite NiFe_2_O_4_ has features that make it ideal for use in hospital wastewater treatment, including enormous adsorption ability, ease of functionality at the lowest cost, with high reactivity and sensitivity, where it proved an effective treatment for pharmaceutical effluent, with a clearance percentage greater than 85%. Nanotechnology is a highly promising field with a bright future for hospital wastewater control in brain protection against neurodegeneration. So, magnetically adsorbent nanoparticles could be further developed or integrated into broader wastewater treatment systems globally.

## Data Availability

No datasets were generated or analysed during the current study.

## Abbreviations

AChE: Acetylcholinesterase
AMX: Amoxicillin
ATP: Adenosine triphosphates
BBB: Blood–brain barrier
b.wt: Body weight
CAT: Catalase
CDNB: 1-Chloro-2,4-dinitrobenzene
CYP2C9: Cytochrome P450 family 2 subfamily C member 9
D1: Dopamine receptor 1
D2: Dopamine receptor 2
DA: Dopamine
DA-ergic: Dopaminergic
DCF: Diclofenac
DOPAC: 3,4-Dihydroxyphenylacetic acid
DTNB: 5,5-Dithiobis-2-nitrobenzoic acid
ELISA: Enzyme-linked immunosorbent assay
FTIR: Fourier transform infrared spectrometer.
GPx: Glutathione peroxidase
GSH: Reduced glutathione
GSK-3β: Glycogen synthase kinase-3beta
GST: Glutathione S-transferase
H&E: Hematoxylin and eosin
HPLC: High-performance liquid chromatography
5-HIAA: 5-Hydroxyindoleacetic acid
5-HT: Serotonin
HWW: Hospital wastewater
IL-17A: Interleukin*-*17A
LCMS: Liquid chromatography–mass spectrometry
LSD: Least significant difference
MAO: Monoamine oxidase
MAO-A: Monoamine oxidase-A
MAO-B: Monoamine oxidase-B
MDA: Malondialdehyde
MWM: Morris Water Maze
NAc: Nucleus accumbens
NAD +: Nicotinamide adenine dinucleotide
NE: Norepinephrine
NF-κB: Nuclear factor-kappa B
NO: Nitric oxide
Nox2: NADPH oxidase
NSAIDs: Non-steroidal anti-inflammatory drugs
PARP1: Poly(ADP-ribose) polymerase1
ROS: Reactive oxygen species
SEM: Scanning electron microscope
SMZ: Sulfamethoxazole
SOD: Superoxide dismutase
SPSS: Statistical Package for the Social Sciences
TBARS: Thiobarbituric acid
TEM: Transmission electron microscope
TNF-α: Tumor necrosis factor-alpha
XRD: X-ray diffraction
